# Anticancer bioactive peptide-3 inhibits human gastric cancer growth by targeting miR-338-5p

**DOI:** 10.1186/s13578-016-0112-8

**Published:** 2016-09-22

**Authors:** Zhiwei Xing, Lan Yu, Xian Li, Xiulan Su

**Affiliations:** 1 Department of Cell Biology, Capital Medical University, Beijing, China; 2 The Inner Mongolia Autonomous Region People’s Hospital, Hohhot, Inner Mongolia China; 3 Clinical Medical Research Center of the Affiliated Hospital, Inner Mongolia Medical University, Hohhot, Inner Mongolia China

**Keywords:** miRNA, Microarray, miR-338-5p, Cisplatin, 5-fluorouracil

## Abstract

**Background:**

Cancer incidence and mortality have been increasing in China, making cancer the leading cause of death since 2010 and a major public health concern in the country. Cancer stem cells have been studied in relation to the treatment of different malignancies, including gastric cancer. Anticancer bioactive peptide-3 (ACBP-3) can induce the apoptosis of gastric cancer stem cells (GCSCs) and reduce their tumorigenicity. In the present study, for the first time, we used a miRNA microarray and bioinformatics analysis to identify differentially expressed miRNAs in ACBP-3-treated GCSCs and GCSC-derived tumors in a xenograft model and functionally verified the identified miRNAs. miR-338-5p was selected based on its significant upregulation by ACBP-3 both in cultured GCSCs and in tumor tissues.

**Results:**

miR-338-5p was downregulated in GCSCs compared with normal gastric epithelial cells, and the ectopic restoration of miR-338-5p expression in GCSCs inhibited cell proliferation and induced apoptosis, which correlated with the upregulation of the pro-apoptotic Bcl-2 proteins BAK and BIM. We also found that ACBP-3-treated GCSCs could respond to lower effective doses of cisplatin (DDP) or 5-fluorouracil (5-FU), possibly because ACBP-3 induced the expression of miR-338-5p and the BAK and BIM proteins and promoted GCSC apoptosis.

**Conclusions:**

Our data indicate that miR-338-5p is part of an important pathway for the inhibition of human gastric cancer stem cell proliferation by ACBP-3 combined with chemotherapeutics. ACBP-3 could suppress GCSC proliferation and lower the required effective dose of cisplatin or 5-fluorouracil. Therefore, this study provides not only further evidence for the remarkable anti-tumor effect of ACBP-3 but also a possible new approach for the development of GCSC-targeting therapies.

**Electronic supplementary material:**

The online version of this article (doi:10.1186/s13578-016-0112-8) contains supplementary material, which is available to authorized users.

## Background

Gastric cancer (GC) is one of the major threats to human health worldwide. More than 60 % of newly diagnosed cases have been detected in Eastern Asia [1]. Cancer incidence and mortality have been increasing in China, making cancer the leading cause of death since 2010 and a major public health concern in the country. It is predicted that there will be approximately 4,292,000 newly diagnosed invasive cancer cases in 2015 in China, corresponding to almost 12,000 new cancer diagnoses on average each day (The total number of cases projected for 2015 are based on the average incidence rates for the most recent 3 years (2009–2011) using data from 72 population-based cancer registries) [2]. It has been suggested that cancer stem cells (CSCs), found in many types of tumors, and may be responsible for cancer relapse and metastasis. Although the CSC population in tumors is very small, CSCs are thought to underlie the malignant phenotype of tumors, including cancer initiation, angiogenesis, and invasiveness, metastasis, and drug resistance [3]. Although new chemotherapeutic, radio therapeutic and surgical methods have been developed to treat GC, the 5-year survival rate has not improved. One of the main reasons is the existence of gastric cancer stem cells (GCSCs), which can resist chemo- and radiotherapy, escape from apoptosis, and proliferate infinitely [4], whereas differentiated GC cells are eliminated. Thus, therapeutic approaches targeting GCSCs in GC have been a focus of recent research.

Natural antitumor agents have attracted considerable attention because of their low toxicity and powerful antitumor activity. Anticancer bioactive peptide-3 (ACBP-3), a polypeptide isolated from goat liver in our laboratory, has been shown to inhibit proliferation, induce apoptosis, and reduce the tumorigenicity of human GCSCs both in vitro and in vivo. In addition, ACBP-3 could lower the required effective dose of DDP and improve the condition of mice bearing GCSC-originating tumors [5]. However, the mechanisms underlying the ACBP-3-mediated suppression of GCSCs remains unknown.

MicroRNAs (miRNAs) are small non-coding RNAs of approximately 22 nucleotides that function by targeting mRNAs and regulating gene expression. miRNAs are involved in multiple physiological and pathological processes [6], including cancer, in which they regulate cell proliferation, differentiation, infiltration, and apoptosis [7, 8]. Thus, miRNA microarrays are widely used as a powerful tool for analyzing the responses of cancer cells to treatments.

**Fig. 1:**
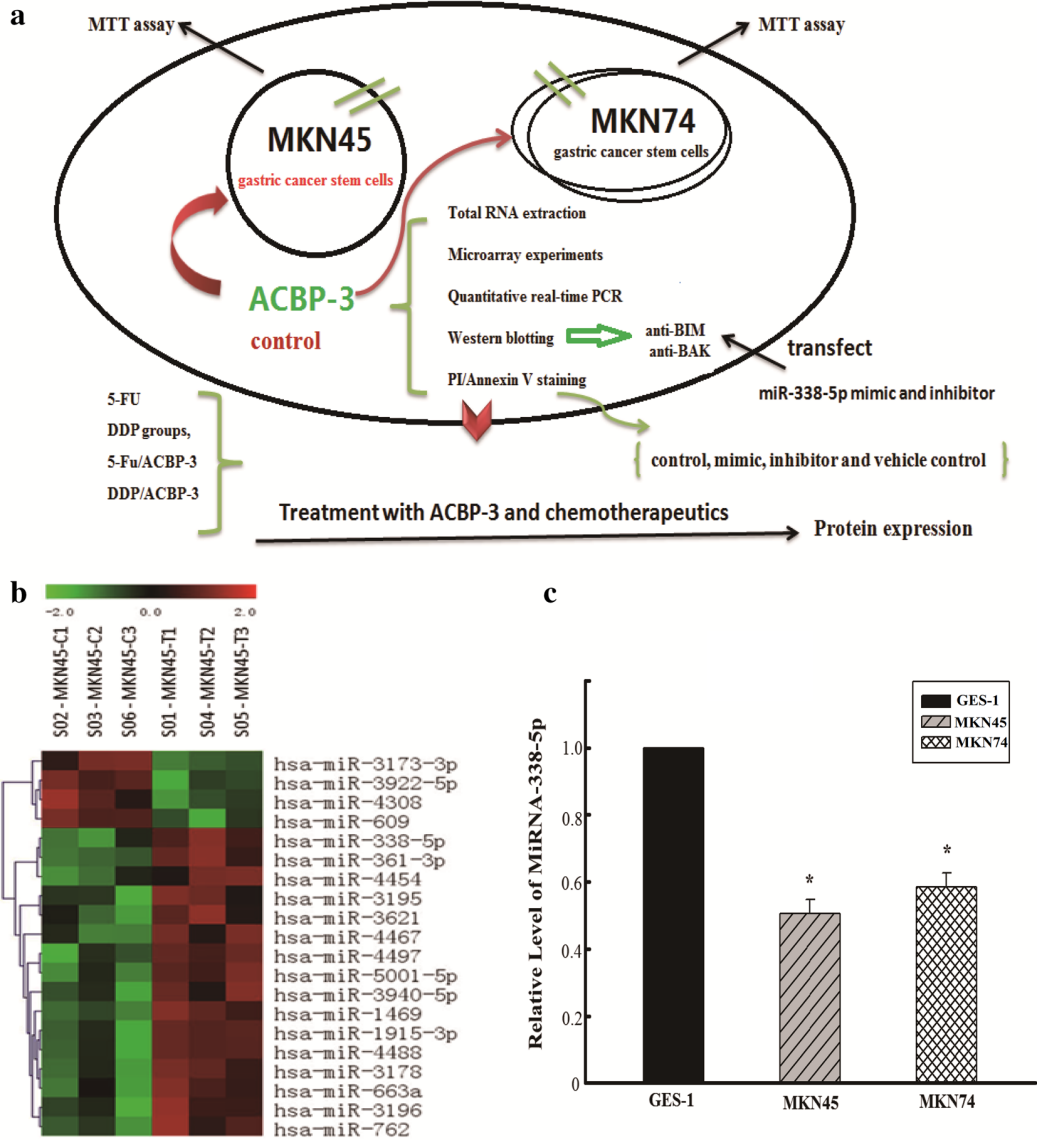
A mechanism of human gastric cancer stem cell inhibition by anticancer bioactive peptide-3 (**a**). Effects of ACBP-3 on miRNA expression in human GCSCs. Twenty and nine significantly differentially expressed miRNAs were identified in cultured GCSCs (**b**) (*P* < 0.05). **c** Down regulation of the relative miR-338-5p expression in GCSCs compared with normal human gastric epithelial cells, (*P < 0.05, compared with gastric epithelial GES-1 cells)

In the present study, we investigated the inhibitory activity of ACBP-3 against GCSCs using a miRNA microarray followed by bioinformatics analysis to screen for miRNAs that are differentially regulated in ACBP-3-treated GCSCs (Fig. 1a). The identified miRNA miR-338-5p was functionally verified in GCSCs exposed to chemotherapeutic drugs or drugs combined with ACBP-3 and were shown to inhibit GCSC proliferation via the upregulation of apoptosis. Thus, in this study, we present a mechanism of ACBP-3 activity against GCSCs that explains the potentiating effect of ACBP-3 on cancer cell inhibition by chemotherapeutic drugs.

## Results

### Selection of miRNA species differentially expressed in ACBP-3-treated GCSCs

The comparison of miRNA expression profiles identified 20 and nine miRNAs in ACBP-3-treated GCSCs and controls, respectively that were significantly differentially expressed (Fig. 1b, c). The differentially expressed miRNAs detected in two chips did not overlap, so, after multiple rescreening processes, nine miRNAs significantly upregulated by ACBP-3 (*P* < 0.05) were selected for further analysis: hsa-miR-1469, hsa-miR-762, hsa-miR-338-5p, hsa-miR-3173-3p, hsa-miR-4467, hsa-miR-3940-5p, hsa-miR-3195, hsa-miR-3621, and hsa-miR-663a (Table 1). To determine the putative biological functions of the selected miRNAs, TargetScan and Mirdb were used to identify 639 miRNA target genes, which were subjected to multiple analyses, including GO analysis (Additional file 1: Figure S1), pathway analysis (Additional file 1: Figure S2), miRNA-gene-network analysis (Additional file 1: Figure S3), and mRNA-GO-network analysis (Additional file 1: Figure S4) to select functionally relevant genes involved in carcinogenesis. In Additional file 1: Figure S3, the miRNA-338-5p, the miRNA—338-3 p and the miRNA-762-5 miRNAs play a key role in the network, especially the miRNA-338-5p regulated most genes in the network, to 131, including the BCL-2 families and KRAS families of related tumor genes. In the microarray results, we observed that the miRNA-3173-3p regulated BAK and the miRNA-338-5p regulated BIM (BCL2L11).

**Table 1:** Nine miRNAs selected after rescreening

miRNA_miRBase-18th	Fold change (3/1)	Direction
hsa-miR-1469	1.262138297	Upregulated
hsa-miR-762	1.200592588	Upregulated
hsa-miR-338-5p	2.129779884	Upregulated
hsa-miR-3173-3p	1.226251386	Upregulated
hsa-miR-4467	1.284049958	Upregulated
hsa-miR-3940-5p	1.336691618	Upregulated
hsa-miR-3195	1.376221162	Upregulated
hsa-miR-3621	1.273270254	Upregulated
hsa-miR-663a	1.221943009	Upregulated

### miR-338-5p expression in GCSCs and normal gastric epithelial cells

As miR-338-5p demonstrated the highest upregulation by ACBP-3, it was selected for validation by qRT-PCR. We compared the relative miR-338-5p expression in GCSCs and normal human gastric epithelial GES-1 cells and found that the miR-338-5p levels were 1.96 and 1.71 times higher in GES-1 cells than in MKN45- and MKN74G-derived GCSCs, respectively (Fig. 1d).

### miR-338-5p-mediated inhibition of GCSC proliferation

**Fig. 2:**
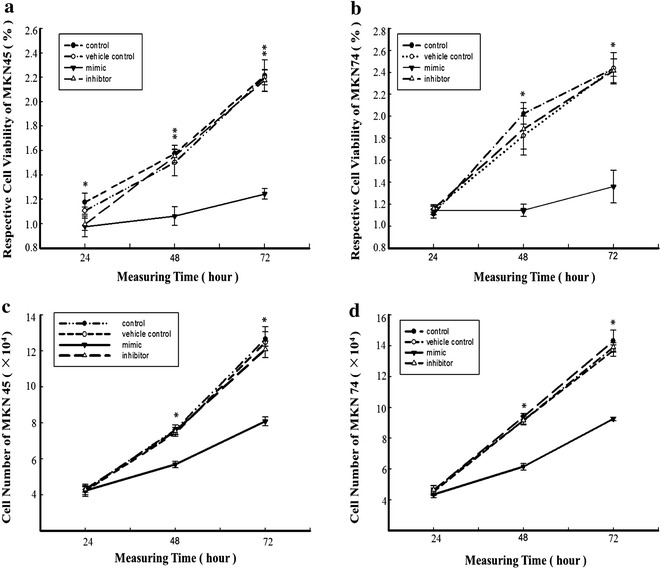
Effect of miR-338-5p expression on GCSC proliferation. Cell proliferation was assessed by the MTT assay in MKN45-derived (**a**, **c**) and MKN74-derived GCSCs (**b**, **d**) 24 h, 48 h, and 72 h after transfection with miR-338-5p mimic or inhibitor. The effect of miR-338-5p expression on GCSC viability was determined by an MTT assay. Viable cells were counted in MKN45 (**c**) and MKN74 GCSCs (**d**) 24 h, 48 h, and 72 h after transfection with miR-338-5p mimic or inhibitor (*Filled circle* represents the control, *open circle* represents the vehicle control, *filled inverted traingle* represents the mimic, *open triangle* represents the inhibitor). *(*P* < 0.05) and **(*P* < 0.01) represent comparisons of two symbols in the same row at a set time

To clarify the functional role of miR-338-5p, its mimic and inhibitor were used to transfect GCSCs. Transfection with miR-338-5p resulted in 19.71-and 28.29-fold upregulation of the miR-338-5p levels in MKN45- and MKN74-derived GCSCs, respectively (*P* < 0.01), whereas the miR-338-5p inhibitor decreased miR-338-5p levels by 58.4 and 39.6 %, respectively, of the control (*P* < 0.01) (Additional file 1: Figure S5 A–D). There was no difference between the controls in each group (*P* > 0.05). The MTT assay indicated that miR-338-5p overexpression inhibited the proliferation of MKN45-derived cells by 16.95 % ± 1.19 (*P* < 0.05), 32.48 % ± 2.26 (*P* < 0.01), and 43.89 % ± 5.72 (*P* < 0.01) at 24, 48, and 72 h post-transfection, respectively, whereas the miR-338-5p inhibitor had no effect (*P* > 0.05) (Fig. 2a). Similar changes were observed in miR-338-5p-transfected MKN74 GCSCs, in which cell proliferation was inhibited to 48.63 % ± 6.03 and 48.21 % ± 4.91 of that in the control cells at 48 and 72 h, respectively (*P* < 0.05; Fig. 2b).

Viable cell counting confirmed the above results. At 48 h post-transfection, the cell numbers decreased to 25.33 % ± 2.14 (*P* < 0.01) and 34.51 % ± 4.02 (*P* < 0.05), and at 72 h, the cell numbers were 36.05 % ± 2.57 (*P* < 0.01) and 35.31 % ± 4.11 (*P* < 0.05) for miR-338-5p-transfected MKN45 and MKN74 GCSCs, respectively, compared with the controls (Fig. 2c, d). However, the miR-338-5p inhibitor did not significantly affect cell viability (*P* > 0.05).

### miR-338-5p induction of GCSC apoptosis

**Fig. 3:**
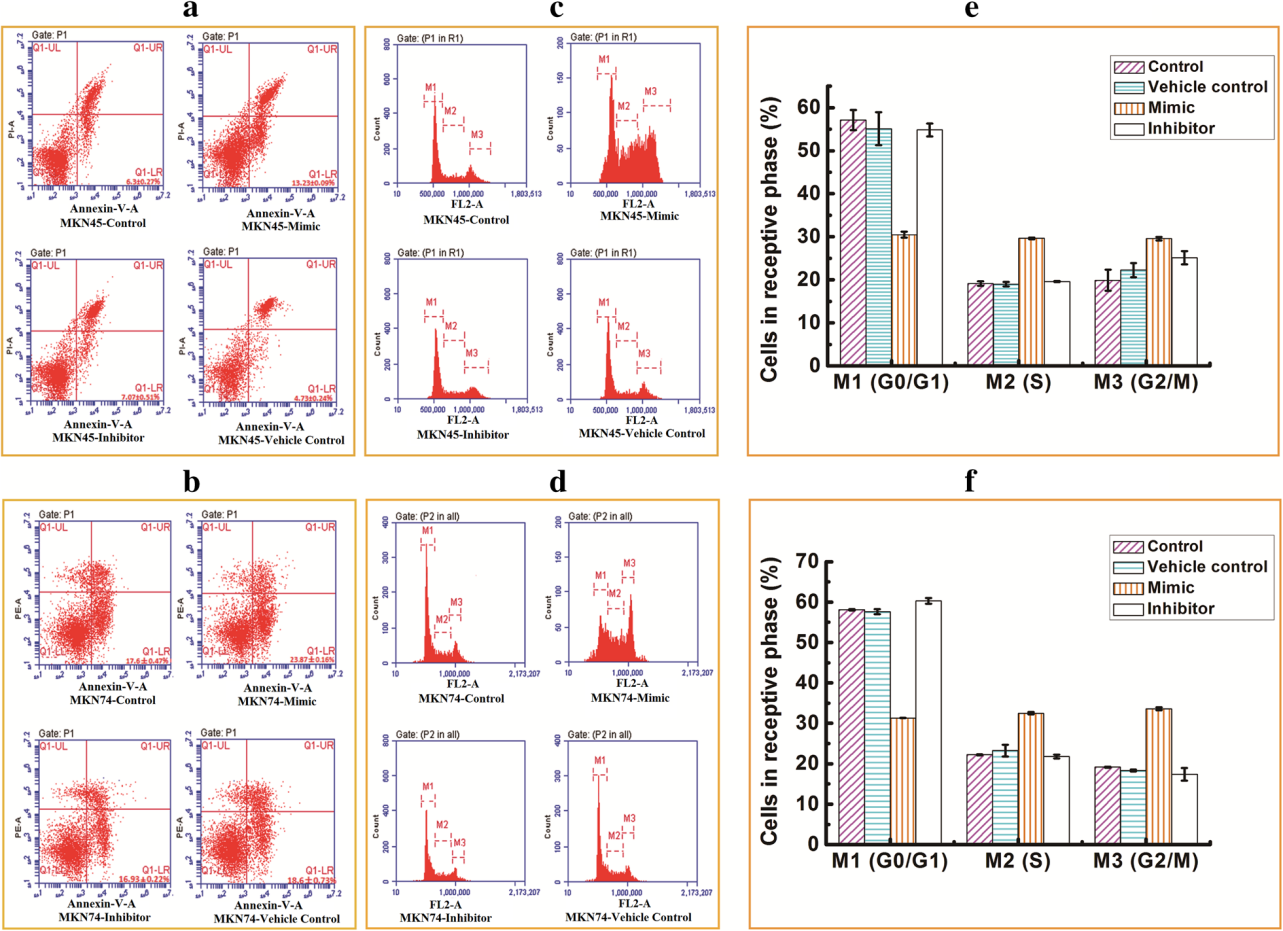
PI/Annexin-V double staining evaluating the effect of miR-338-5p expression on apoptosis and the cell cycle in GCSCs. Untreated control cells and cells treated with a mimic, inhibitor or vehicle control were evaluated for MKN45 cells (**a**, **c**) and MKN74 cells (**b**, **d**) by flow cytometry. The cell cycle phase distribution of MKN45-derived GCSCs (**e**) and MKN74-derived GCSCs (**f**) is described 48 h post-transfection

To evaluate the effects of miR-338-5p on the apoptosis of GCSCs, transfected GCSCs were analyzed by Annexin V-PI staining. There was a significant difference in apoptosis between the control and miR-338-5p-transfected MKN45-derived GCSCs and MKN74-derived GCSCs (Fig. 3a, b). No difference in apoptosis was observed between the control and miR-338-5p inhibitor-transfected GCSCs (*P* > 0.05). Figure 3a shows that the apoptosis rate of miR-338-5p-transfected MKN45-derived GCSCs was increased compared with control cells (13.23 ± 0.09 vs 3 ± 0.27 %, respectively; *P* < 0.05). Similarly, Fig. 3b shows that apoptosis was increased in miR-338-5p-transfected MKN74-derived GCSCs (23.87 ± 0.16 % vs 17.6 ± 0.45 in the control). As in the previous experiments, transfection with the miR-338-5p inhibitor had no effect (*P* > 0.05).

### miR-338-5p deregulation of the cell cycle

The effects of miR-338-5p on the cell cycle were assessed in GCSCs 48 h post-transfection by flow cytometry after Annexin V-PI staining. The results indicate that miR-338-5p decreased the proportion of GCSCs in the G0/G1 phase and increased the proportion in the S and G2/M phases (*P* < 0.01), whereas the miR-338-5p inhibitor did not cause significant cell cycle changes in GCSCs (Fig. 3c–f). Another result was reported in a recent study, in which the transfection of miR-199a-5p inhibitors increased the expression of CTGF and promoted the viability of the cells by increasing the fraction of cells in the G2/M and S phases [9]. Another study reported the Cadmium (Cd)-induced reduction of the number of G0/G1 phase cells and an increase in the number of cells in S phase and G2/M phase, which showed that Cd-induced ROS formation promoted HMGA2 upregulation, causing changes in the cell cycle [10]. Therefore, the over-expression of miR-338-5p decreased the number of GCSCs in G0/G1 phase and increased the number of GCSCs in S and G2/M phase, causing changes in the cell cycle changes and making the cells more likely to be inhibited by chemotherapy drugs. Thus, miR-338-5p, as an anti-cancer gene, can inhibit GCSC proliferation.

**Fig. 4:**
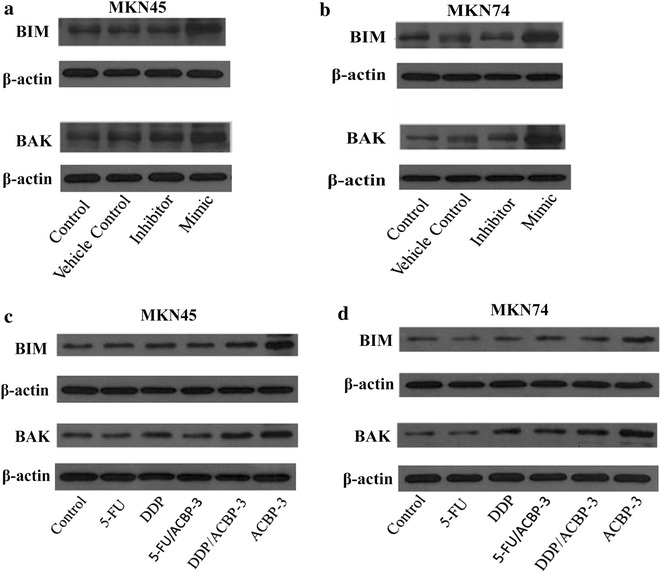
Correlation between miR-338-5p expression and the relative level of BIM and BAK. BIM and BAK expression was analyzed by western blotting in GCSCs transfected with miR-338-5p mimic and inhibitor. Representative blots are shown for miR-338-5p overexpression experiments in transfected MKN45 (**a**) and MKN74 GCSCs (**b**). β-actin was used as a reference. The protein expression of BIM and BAK is shown for GCSCs treated with 5-FU, DDP groups, 5-Fu/ACBP-3, DDP/ACBP-3, ACBP-3 or a control for 48 h. Transfected MKN45 cells (**c**) and MKN74 cells (**d**) are shown, and β-actin was used as a reference

In a recent study, the BH3-only protein BIM, a key post-transcriptional repressor of BIM expression, and the activation of BAX/BAK were shown to be dependent on mitochondrial (intrinsic) apoptosis [11]. Microarray results predicted that mir-338-5p is regulated by a target gene network consisting of 2 closely related genes, namely BAK and BIM (BCL2L11), directly or indirectly [12]. To verify the induction of apoptosis by miR-338-5p in GCSCs, the expression of the pro-apoptotic proteins BIM and BAK was measured by western blotting (Fig. 4a, b). β-actin was used as a reference for the relative level of BIM (Additional file 1: Figure S6 A, C) and BAK (Additional file 1: Figure S6 B, D) in transfected MKN45 cells (Additional file 1: Figure S6 A, B) and MKN74 cells (Additional file 1: Figure S6 C, D). The data indicated that the BIM and BAK levels were increased in both MKN45- and MKN74-derived GCSCs after transfection with miR-338-5p compared with control GCSCs (*P* < 0.05; Fig. 4a, b), confirming the results of flow cytometry.

### Expression of BIM and BAK in GCSCs treated with different drug combinations

Given that miR-338-5p overexpression accelerated GCSC apoptosis and that ACBP-3 (alone or in combination with chemotherapeutic drugs) potently upregulated miR-338-5p levels, we investigated the expression of the pro-apoptotic proteins BIM and BAK in GCSCs treated with ACBP-3, DDP and 5-FU, alone or in combination. The protein expression of BIM and BAK in GCSCs treated with 5-FU, DDP groups, 5-Fu/ACBP-3, DDP/ACBP-3, ACBP-3 or a control treatment for 48 h is shown in Fig. 4c, d. β-actin was used as a reference for the relative level of BIM (Additional file 1: Figure S6 E, G) and BAK (Additional file 1: Figure S6 F, H) in MKN45 cells (Additional file 1: Figure S6 E, F) and MKN74 cells (Additional file 1: Figure S6 G, H). The results showed that in GCSCs, the expression of BIM and BAK positively correlated with that of miR-338-5p. Thus, the BIM and BAK levels were the highest after treatment with ACBP-3, followed by its combination with chemotherapeutic drugs and 5-FU or DDP alone. These results suggest that miR-338-5p could play an important role in the anti-GCSC effects of ACBP-3 by inducing GCSC apoptosis via the upregulation of BIM and BAK expression.

### Determination of experimental doses for chemotherapeutic drugs

**Fig. 5:**
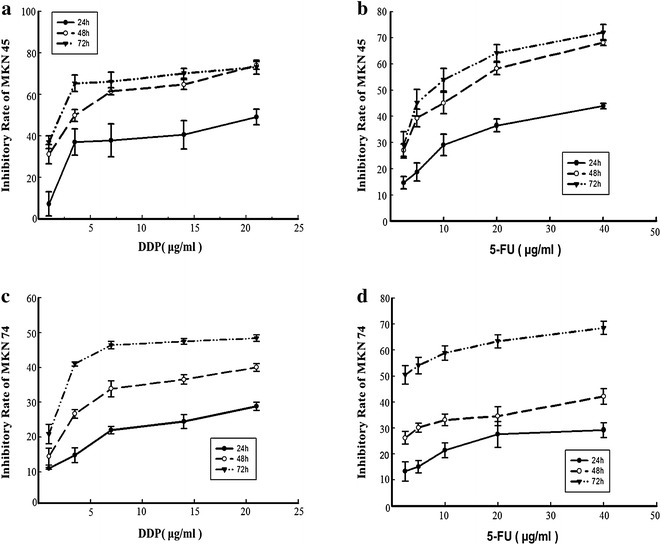
Inhibition of cell proliferation. Inhibition of MKN45-derived (**a**, **b**) and MKN74-derived (**c**, **d**) GCSC proliferation by DDP (**a**, **c**) and 5-FU (**b**, **d**). Cell proliferation was determined by an MTT assay after 24, 48, and 72 h of exposure to different doses of the chemotherapeutic agents

**Fig. 6:**
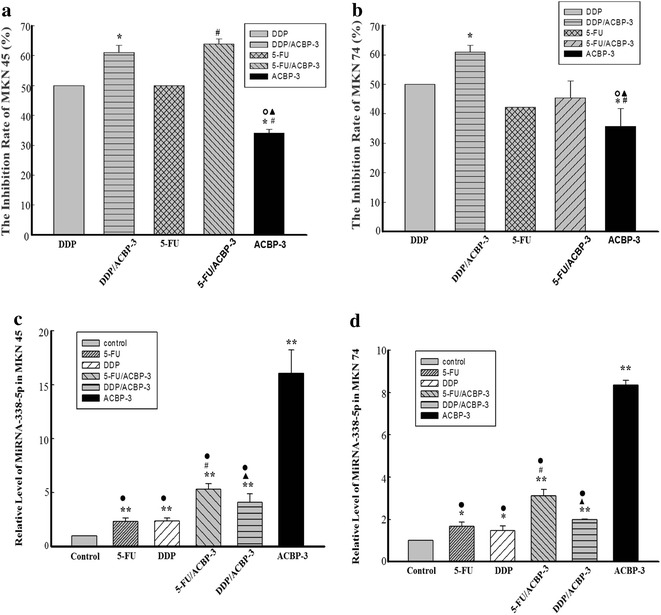
Inhibition of GCSC proliferation by a combination of chemotherapeutic drugs and ACBP-3. MKN45-derived GCSCs (**a**); MKN74-derived GCSCs (**b**).* Asterisks* representing DDP/ACBP-3 and ACBP-3 vs DDP, *P* < 0.01;* hash symbol* representing ACBP-3 vs 5-FU, *P* < 0.01; *open circle* and *filled triangle* representing ACBP-3 vs DDP/ACBP-3 and 5-FU/ACBP-3, *P* < 0.01, respectively. Relative miR-338-5p expression in GCSCs treated with ACBP-3 and chemotherapeutic drugs. GCSCs derived from MKN45 (**c**) and MKN74 (**d**) were treated with ACBP-3 alone or in combination with 5-FU or DDP for 48 h and analyzed for miR-338-5p expression. *P < 0.05, **P < 0.01, 5-FU/ACBP-3 vs 5-FU; *filled traingle* representing DDP/ACBP-3 vs DDP, *P* < 0.01; *filled circle* representing ACBP-3 vs other treatments, *P* < 0.01

ACBP-3 could lower the required effective dose of DDP, enhance its therapeutic efficiency, and improve the survival of tumor-bearing mice [5]. Here, we used a combination of ACBP-3 with DDP and 5-FU to analyze the effects on GCSCs in vitro. To determine the IC_50_ for DDP and 5-FU in GCSCs, the cells were treated with increasing doses of DDP and 5-FU for 24, 48, or 72 h, and the IR was calculated. Both DDP and 5-FU inhibited GCSCs in a dose-and time-dependent manner. Thus, for MKN45-derived GCSCs, the IR increased from 7.16 ± 0.06 % at 1 μg/ml DDP to 48.99 ± 0.04 % at 21 μg/ml DDP after 24 h of treatment, and from 36.91 ± 0.06 % at 24 h to 49.80 ± 0.03 % at 48 h and 65.25 ± 0.04 % at 72 h for 3.5 μg/ml DDP (*P* < 0.01; Fig. 5). Similar dynamics were observed for 5 μg/ml of 5-FU (Fig. 5). Based on these data, the IC_50_ was calculated (Table 2). However, a linear increase in the IR was observed only within a certain drug concentration range and disappeared at higher doses (Fig. 6a, b). Thus, 199.10 μg/ml 5-FU caused 50 % inhibition (IC_50_) of MKN74-derived GCSCs, whereas a significantly lower dose (40 μg/ml) caused 42.16 % inhibition. Therefore, we chose 40 μg/ml 5-FU for MKN74-derived GCSCs; in other cases, the IC_50_ was used. Both MKN45- and MKN74-derived GCSCs treated with different drug doses for 48 h remained stable; therefore, we chose 48 h as the treatment period in further experiments.

**Table 2:** IC_50_ values of chemotherapeutic drugs for MKN45-derived GCSCs (μg/mg) and MKN74-derived GCSCs (μg/ml)

	24 h	48 h	72 h
*MKN45*			
DDP	12.72	3.85	1.75
5-FU	111.43	11.87	8.28
*MKN74*			
DDP	30	10.62	3.16
5-FU	313.87	199.10	2.57

### ACBP-3 potentiation of DDP and 5-FU cytotoxicity for GCSCs

GCSCs were treated with 5-FU and DDP at a concentration of 0.5 IC_50_ alone or in combination with 22 μg/ml ACBP-3 [5] and analyzed for the IR. The results showed that in MKN45-derived GCSCs, both drugs combined with ACBP-3 caused significantly stronger inhibition of cell proliferation than when the drugs were used alone (*P* < 0.01; Fig. 6a, b), whereas in MKN74-derived GCSCs, DDP in combination with ACBP-3 inhibited cell proliferation more effectively than when the drug was used alone (*P* < 0.01; Fig. 6a, b). However, the combination of 5-FU with ACBP-3 had the same effect on MKN74-derived GCSCs as 5-FU alone, although it was higher than that of ACBP-3 alone (*P* < 0.01).

### Relative miR-338-5p expression in GCSCs treated with different drug combinations

The above results confirmed that ACBP-3 potentiated the anti-GCSC activity of DDP and 5-FU, which is consistent with the findings in vivo [5]. To address the mechanism underlying the observed synergy, we investigated miR-338-5p expression in drug-treated GCSCs. The results indicated that in all treated MKN45- and MKN74-derived GCSCs, miR-338-5p expression was higher compared with the control (*P* < 0.01 and *P* < 0.05, respectively). ACBP-3 alone increased the miR-338-5p levels more than 10 times compared with the control (*P* < 0.01) and significantly elevated the miR-338-5p levels in combination with 5-FU or DDP compared with the drugs alone (*P* < 0.01; Fig. 6c, d). These results suggest that miR-338-5p upregulation by ACBP-3 may be the mechanism responsible for the ACBP-3-mediated potentiation of the cytotoxic effects of drugs against GCSCs.

## Discussion

CSCs can self-renew for a long time and generate differentiated cancer cell progeny [13] because of impaired cell cycle checkpoint control and apoptosis regulation as well as decreased DNA sensitivity to chemotherapy and radiotherapy effects due to the upregulation of multidrug resistance proteins [3]. CSCs can divide asymmetrically to generate differentiated cancer cells or symmetrically to maintain a stable population of their own, which accounts for their resistance to cytotoxic drugs [14, 15] and radiation [16]. A sudden decrease in tumor size due to radical treatments may change the microenvironment and activate CSCs [17], leading to tumor relapse and metastasis [18]. Therefore, effective CSC-targeting therapies would significantly improve cancer prognosis.

Using MKN45 and MKN74 cells, which have the highest GCSC population among human GC cell lines [19], we have found that ACBP-3 exerted GCSC-suppressive effects in vitro and in vivo. Here, we observed that miR-338-5p was highly upregulated by ACBP-3 in cultured GCSCs and GCSC-derived tumors. miR-338 is considered a central nervous system-specific miRNA encoded in the intron of the *AATK* (Apoptosis-associated Tyrosine Kinase) gene [20], which is upregulated during the apoptosis of myeloid precursor cells [21], and cerebellar granule neurons cultured in low-potassium conditions [22]. In miRNA biogenesis, one strand is loaded in the RNA-induced silencing complex, whereas the other is destroyed [23]; but for miR-338, both miR-338-3p and miR-338-5p work as transcriptional repressors [24–26]. Previous studies have indicated that miR-338 and miR-338-3p are downregulated in gastric cancer [27, 28], esophageal squamous carcinoma [29, 30], hepatocellular carcinoma [31, 32], and neuroblastoma [33] and upregulated in oral cancer [34], pancreatic cancer [35], and osteosarcoma [36], suggesting cancer type-specific effects. However, the activity of miR-338-5p has been rarely reported, and the functional relationship between miR-338-3p and miR-338-5p remains unclear.

The present study revealed that miR-338-5p was downregulated in GCSCs compared with normal gastric epithelial cells, suggesting that miR-338-5p may function as an inhibitor of GC development. Given that ACBP-3 induced miR-338-5p in GCSCs and GCSC-initiated tumors, the inhibitory effect of ACBP-3 on GCSCs could be due to the upregulation of miR-338-5p expression. ACBP-3 also inhibited proliferation and promoted apoptosis in GCSCs, which directly correlated with miR-338-5p upregulation, as demonstrated in miR-338-5p-transfected GCSCs [36]. However, a miR-338-5p inhibitor did not show any effects, probably because the starting level of miR-338-5p expression was already downregulated to the baseline and could not be functionally inhibited any further.

Interestingly, in contrast to cell proliferation and viability inhibition, a miR-338-5p mimic induced the transition of GCSCs to the S or G2/M phase of the cell cycle, i.e., accelerated GCSC division. GCSCs have considerable resistance to chemotherapeutic agents due to their relative quiescence and secluded location. It is possible that miR-338-5p facilitates GCSC division and conversion into differentiated GC cells, which are more susceptible to chemotherapy, thus changing quiescent GCSCs to a more vulnerable state. miR-338-5p may first induce GCSCs to divide and differentiate and then promote their apoptosis via unrelated mechanisms. In previous studies, GCSCs were found to be enriched in CD44^+^ MKN45 and MKN74 cells [5, 19], whereas ACBP-3 could decrease the ratio of CD44^+^/CD44^−^ cells, which provides support for our hypothesized mechanism of miR-338-5p activity. Further experiments are required to test these speculations.

BAK and BIM are pro-apoptotic members of the BCL-2 family, which controls the mitochondrial apoptotic pathway [37]. The results indicated that BAK and BIM levels correlated with miR-338-5p expression in GCSCs, suggesting that these proteins may be induced by ACBP-3 via miR-338-5p upregulation and then trigger the apoptosis of GCSCs. This mechanism can provide an explanation for the anticancer effects of ACBP-3 related to GCSC apoptosis [5].

DDP and 5-FU are representative platinum-based and fluorouracil-based chemotherapeutic drugs widely used as first-line anti-cancer agents in the clinic. However, they exert severe side effects, including myeloid suppression, hepatotoxicity, ototoxicity, gastrointestinal toxicity, nephrotoxicity, and peripheral neuropathy [38], which are exacerbated if different drugs are combined. Our previous results indicated that ACBP-3 could lower the effective dose of DDP [5] and improve the quality of life of tumor-bearing mice [5, 39]. In this study, we observed that the combination of ACBP-3 with DDP and 5-FU at 0.5 IC_50_ resulted in stronger inhibition of GCSC proliferation compared with the drugs alone; in addition, the potentiating effect correlated with miR-338-5p expression. As our results indicate, the anticancer efficiency of DDP or 5-FU, which is much stronger than that of ACBP-3, does not depend on miR-338-5p expression, suggesting other cancer-suppressive mechanisms, which may not be sufficient to overcome GCSC resistance. Therefore, the combination of these mechanisms and miR-338-5p upregulation exerted by ACBP-3 may be efficient against GCSCs.

ACBP-3 is a natural compound with low in vivo cytotoxicity [5]. Combined with strong anti-cancer chemotherapeutic agents, it may lower their effective dose by increasing miR-338-5p levels in GCSCs and increasing their sensitivity to chemotherapy while reducing the side effects of the drugs. In a previous study, we conducted a preliminary analysis of the composition of ACBP-3 [5]. In the future, we plan to identify each component and perform detailed functional evaluation, as well as provide further mechanistic insights into ACBP-3 activity.

## Conclusions

Our results show, for the first time, that miR-338-5p plays an important role in the inhibition of human gastric cancer stem cell proliferation by ACBP-3 combined with chemotherapeutics. ACBP-3 upregulated miR-338-5p expression, increased BAK and BIM levels, promoted apoptosis and inhibited the proliferation of GCSCs. Our results suggest that ACBP-3 could lower the effective dose of DDP or 5-FU, providing a new direction for CSC-targeting therapy.

## Methods

### ACBP-3 production

ACBP-3 was isolated from goat liver as previously described [40].

### Isolation of GCSCs from human GC cells

GC MKN45 cells were purchased from the Cell Resource Center, Institute of Basic Medical Sciences, Chinese Academy of Medical Sciences, Peking Union Medical College (Beijing, China). MKN74 and GES-1 cells were a gift from the laboratory of Prof. Yang K (Health Center at the Peking University College of Medicine, Beijing, China). The cells were cultured in RPMI-1640 (Gibco BRL, Gaithersburg, MD, USA) with 1 % penicillin–streptomycin and 10 % fetal bovine serum (FBS; JRH Biosciences, Lenexa, KS, USA) at 37 °C in a humidified 5 % CO_2_ incubator. Spheroid colony-derived cells, which constitute a very small proportion of MKN45 and MKN74 GC cells, were identified as GCSCs according to a published method [5]. GCSCs from MKN45 cells were treated with 22 μg/ml ACBP-3 or the same volume of PBS (control). The authenticity of these cell lines was confirmed by the Cell Resource Center, Institute of Basic Medical Sciences, Chinese Academy of Medical Sciences, Peking Union Medical College. No authentication of these cell lines was performed by the authors.

### Total RNA extraction

Total RNA was extracted from the treated GCSCs and tumor tissues using Trizol (Invitrogen, Paisley, UK) according to the manufacturer’s instructions. RNA concentration was determined using a NanoDrop 2000 spectrophotometer (Thermo Scientific, Wilmington, DE, USA), and its integrity was confirmed by denaturing gel electrophoresis.

### Microarray experiments

miRNA expression profiling was performed using Affymetrix GeneChip miRNA 3.0 arrays (Affymetrix, Santa Clara, CA, USA) containing 19,931 human mature miRNAs in miRBase 17 (http://www.mirbase.org). miRNA expression data are available from the NCBI Gene expression omnibus (GEO). Data processing including gene ontology (GO) analysis [41], pathway analysis [42, 43], miRNA-gene-network analysis [44–46] and miRNA-GO-network analysis was performed using the Affymetrix Expression Console software (version 1.2).

### Quantitative real-time PCR

Selected miRNAs were validated by qRT-PCR. Specific miRNA primers were provided by TIANGEN Biotech (Beijing) Co., Ltd. (Beijing, China), and qRT-PCR was performed using SYBR-Green PCR Master Mix (Takara) and a MX3000P qRT-PCR system (Strata gene) according to the manufacturer’s recommendations. U6 small nuclear RNA was used as a reference gene for normalization. Relative miRNA expression was calculated using the Δ_CT_ method [47].

### Overexpression and inhibition of the target miRNA in GCSCs

GCSCs isolated from MKN45 and MKN74 cells were seeded in 96-well plates at 3 × 10^5^ cells/well in antibiotic-free Opti-MEM supplemented with 10 % FBS and transiently transfected with miR-338-5p mimic or its inhibitor (RIBOBIO, Guangzhou, China) using Lipofectamine 2000 (Thermo Fisher Scientific, Waltham, MA, USA) according to the manufacturer’s instructions; vector-transfected cells served as a control. After 24 h of incubation at 37 °C in a humidified 5 % CO_2_ incubator, the cells were changed into fresh complete medium and cultured for different times.

### GCSC proliferation, apoptosis, and cell cycle analysis

After GCSCs were transfected for 24, 48, and 72 h, 1-(4, 5-dimethylthiazol-2-yl)-3, 5-diphenylformazan thiazolyl blue formazan (MTT formazan; Sigma, St. Louis, MO, USA) was added to the wells and incubated at 37 °C for 5 h. Then, 150 μl DMSO was added to each well; the 96-well plates were rotated for 10 min to dissolve the purple crystals, and the absorbance at 490 nm was measured using an ELISA plate reader (Biotek Instruments, Highland Park, VT, USA). Apoptosis and the cell cycle were analyzed in GCSCs transfected for 48 h using the Annexin V Apoptosis Detection Kit (eBioscience, San Diego, CA, USA) and ribonuclease (Worthington Biochemical Corp, Lakewood, NJ, USA) according to the manufacturer’s instructions using a flow cytometer (BD Biosciences, Franklin Lakes, NJ, USA).

### Western blot detection of apoptotic proteins

GCSCs transfected with miRNAs or treated with drugs were harvested, lysed with lysis buffer (1 % Triton X-100, 1 mM EGTA, 0.5 % NP-40, 10 mM HEPES (pH 7.4), 1 mM EDTA, 0.15 M NaCl) supplemented with protease inhibitor cocktail (Cell Signaling Technology, Beverly, MA, USA), and sonicated on ice for 10 s. After incubation on ice for 30 min, the lysate was centrifuged at 13,000 rpm for 15 min at 4 °C, the supernatant was collected, and the protein concentration was determined using the Bicinchoninic Acid (BCA) Protein Assay Kit (Pierce, Rockford, IL, USA). Then, 50 g of each lysate was boiled in 5× loading buffer (50 % glycerol, 1 M Tris–HCl, pH 6.8, 10 % β-mercaptoethanol, 10 % SDS, 1 % bromophenol blue), cooled on ice, and separated by SDS-PAGE in 12 % gels. The resolved proteins were analyzed by western blotting using anti-BIM and anti-BAK antibodies (Abcam, Cambridge, MA, USA) and their corresponding secondary antibodies.

### Dose determination for chemotherapeutic drugs

GCSCs were seeded in complete medium in 96-well plates in sextuplicate for 24 h and then treated with 2.5–60 μg/ml 5-FU (Jinyao Pharmaceutical Co., Ltd, Tianjin, China) or 1–21 μg/ml DDP (Haosen Pharmaceutical Co., Ltd., Jiangsu, China). Cultures treated with normal saline served as a control. The cell viability was determined after 24, 48, 72 h by the MTT method, and the absorbance at 490 nm was measured using an ELISA plate reader. The inhibition rate (IR) was calculated as (1−A/B) × 100 %, where A and B are the absorbance values of drug-treated and control cells, respectively, and was used to determine the IC_50_ for each drug.

### Statistical analysis

We used the random-variance model (RVM) *t* test to compare two experimental groups and the RVM F test to compare more than two groups. The RVM model is used to analyze microarray data and screen for differentially expressed genes because it effectively raises the degrees of freedom for small samples. After the significance and false discovery rate (FDR) analyses, we selected differentially expressed genes according to a *P* value considered significant at less than 0.05 [48–50].

## Additional file

10.1186/s13578-016-0112-8 Additional data.

## Abbreviations

ACBP-3: anticancer bioactive peptide-3
GCSCs: gastric cancer stem cells
GC: gastric cancer
CSCs: cancer stem cells
DDP: cisplatin
(5-FU): 5-fluorouracil
miRNAs: MicroRNAs
RVM: random-variance model
FDR: false discovery rate
