# Supplementary material for: Anticancer bioactive peptide-3 inhibits human gastric cancer growth by targeting miR-338-5p
Source: Cell Biosci. 2016 Sep 22;6:53. doi: 10.1186/s13578-016-0112-8 (PMC5034486; doi:10.1186/s13578-016-0112-8)
Supplement: Supplementary file 1 — 10.1186/s13578-016-0112-8 Additional data. [file 13578_2016_112_MOESM1_ESM.docx]

**Supplementary data**

**Figure S1**. **GO analysis.** The vertical axis shows target gene function, the horizontal axis shows enrichment.

**Figure S2**：**Pathway analysis.** The vertical axis shows the pathway of a target gene, the horizontal axis shows enrichment.

**
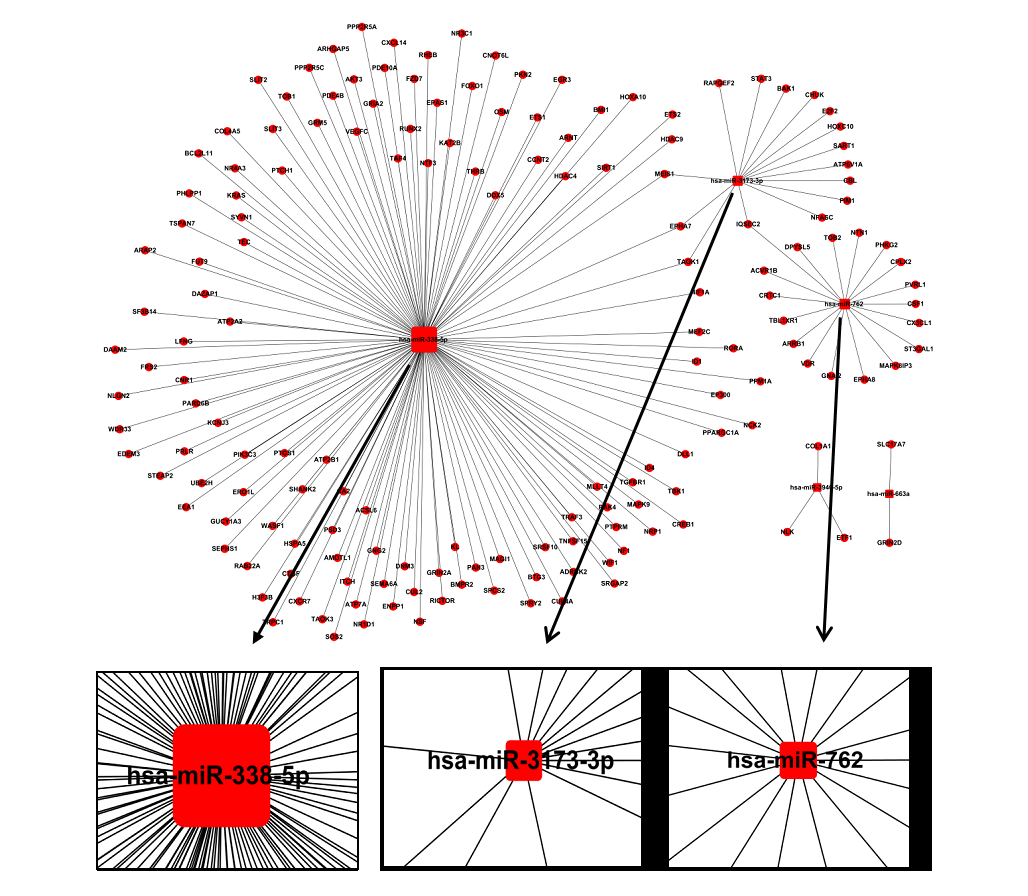
**

**Figure S3**：**The network was constructed based on miRNA-gene relationship.** Rectangles and circles represent miRNAs and genes, respectively.


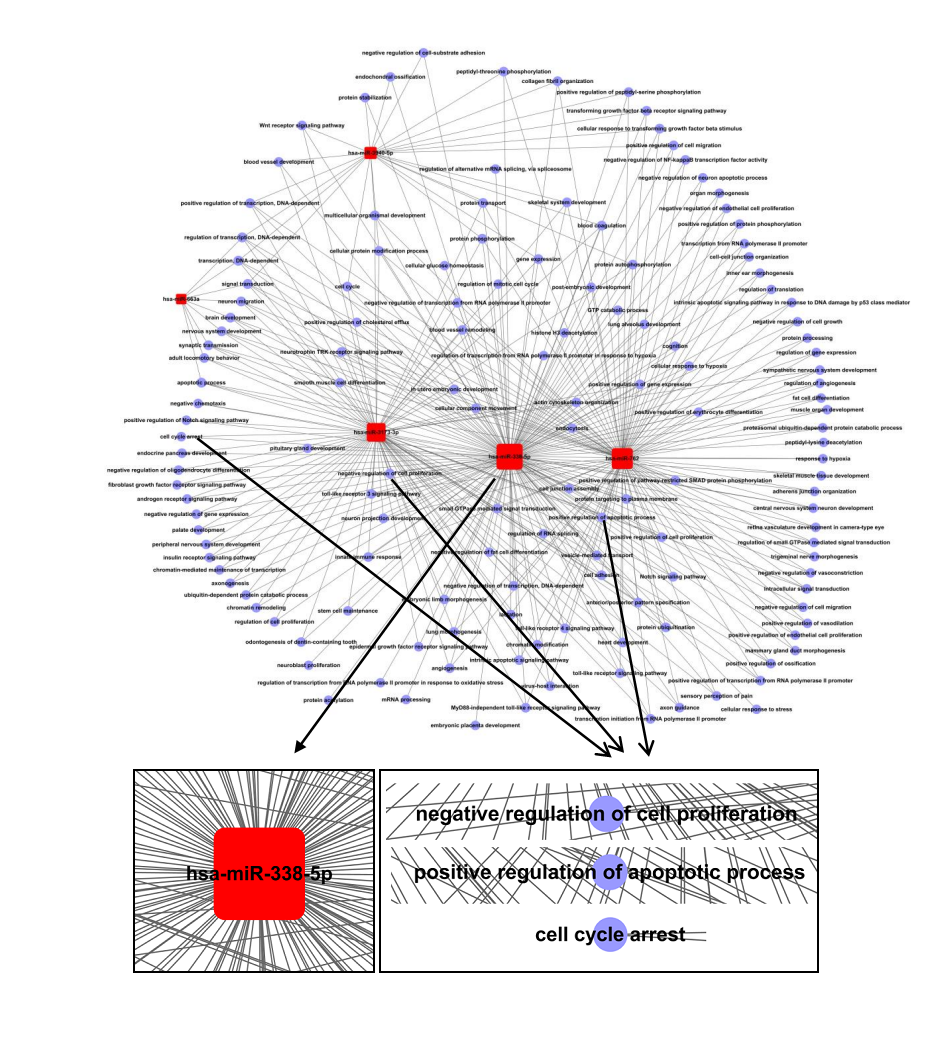


**Figure S4**. **The miRNA- GO map was constructed based on the microarray data.** Rectangles and circles represent miRNAs and GO categories, respectively.


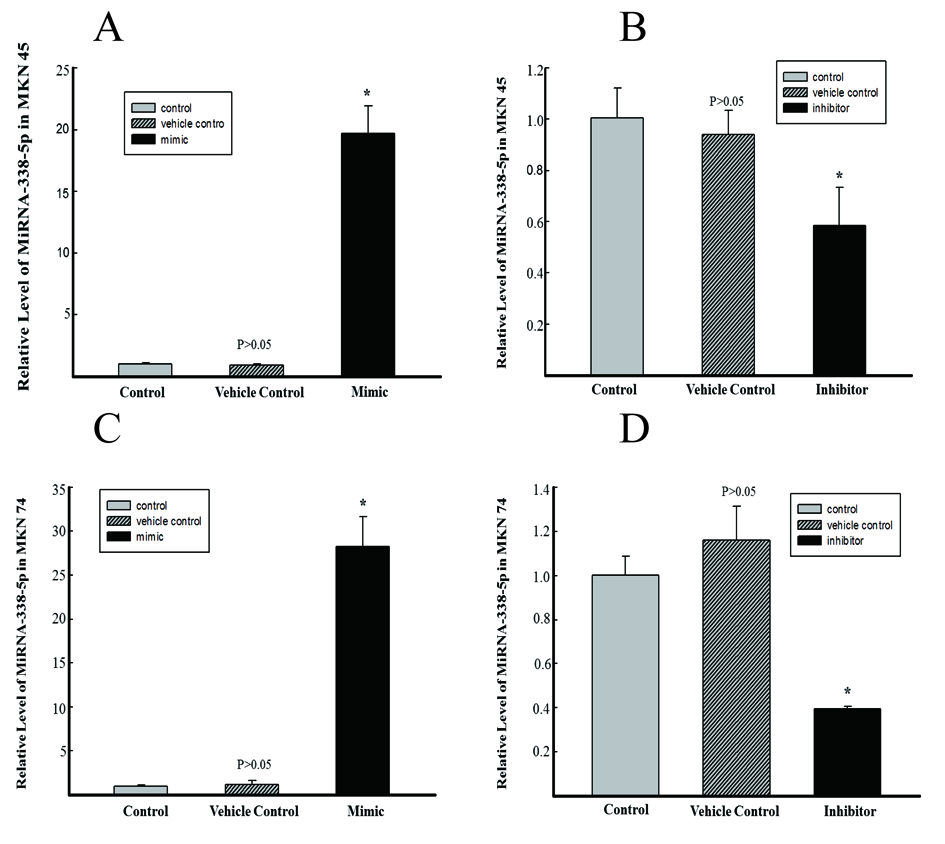


**Figure S5**. **MiR-338-5p expression in GCSCs transfected with miR-338-5p mimic and inhibitor**. MKN45-derived GCSCs (A and B); MKN74-derived GCSCs (C and D). In the miR-338-5p mimic group, miR-338-5p expression increased by 19.7 ± 2.22 and 28.29 ± 3.37 fold, while in the inhibitor group, it decreased to 0.58 ± 0.15 and 0.40 ± 0.01 for MKN45- and MKN74-derived GCSCs, respectively (* *P* < 0.01 versus control).


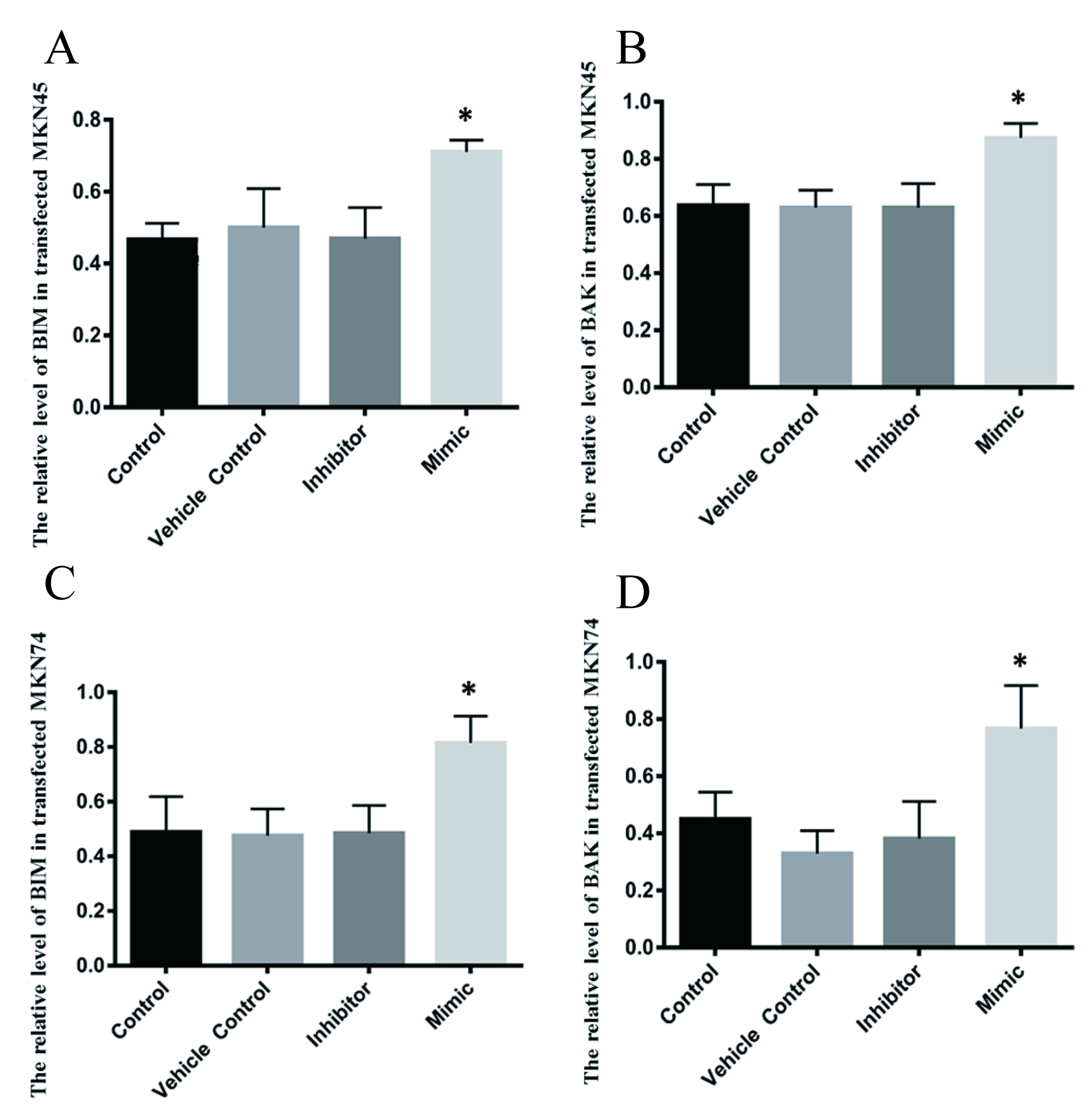


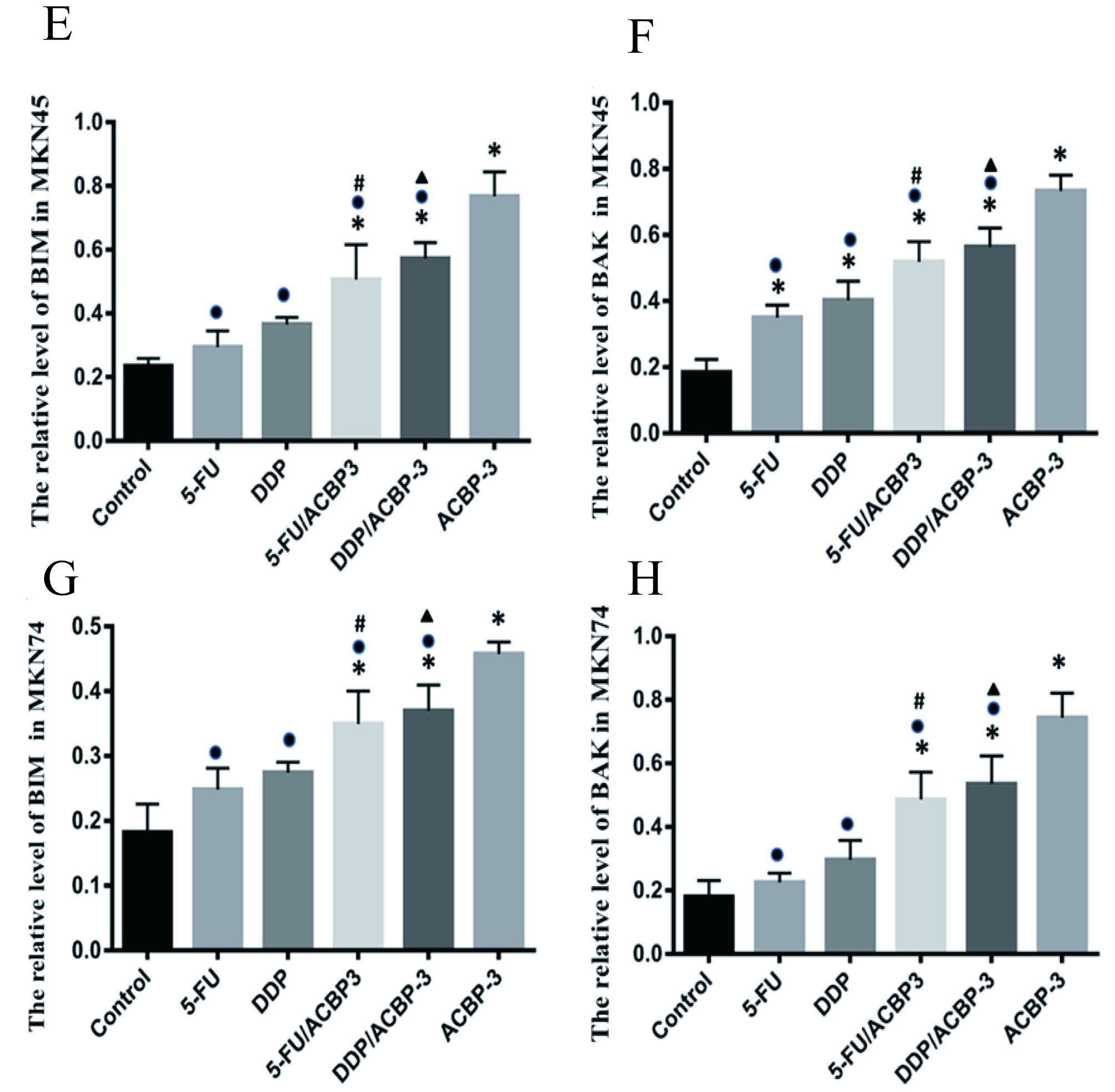


**Figure S6**. **Correlation between miR-338-5p expression and the relative level of BIM and BAK.** BIM and BAK expression was analyzed by western blotting in GCSCs transfected with miR-338-5p mimic and inhibitor, the relative level of BIM (A and C) and BAK (B and D) in transfected MKN45 cells (A and B) and MKN74 cells (C and D), β-actin used as a reference. Protein expression of BIM and BAK in GCSCs treated with 5-FU, DDP groups, 5-Fu/ACBP-3, DDP/ACBP-3, ACBP-3 and control for 48 h, the relative level of BIM (E and G) and BAK (F and H) in MKN45 cells (E and F) and MKN74 cells (G and H), β-actin used as a reference. *, (representing treatment versus control), *P* < 0.05; # (representing 5-FU/ACBP-3 versus 5-FU), *P* < 0.05; ▲ (representing DDP/ACBP-3 versus DDP), *P* < 0.05; and ● (representing each treatment versus ACBP-3), *P* < 0.05.
